# ZooMS confirms geometric morphometrics species identification of ancient sheep and goat

**DOI:** 10.1098/rsos.230672

**Published:** 2023-09-27

**Authors:** Marine Jeanjean, Krista McGrath, Silvia Valenzuela-Lamas, Ariadna Nieto-Espinet, Renate Schafberg, Pere Miquel Parés-Casanova, Sergio Jiménez-Manchón, Claude Guintard, Faiza Tekkouk, Rania Ridouh, Cyprien Mureau, Allowen Evin

**Affiliations:** ^1^ Institute of Evolutionary Science-Montpellier (ISEM), University of Montpellier, CNRS, EPHE, IRD, Montpellier, France; ^2^ Department of Prehistory & Institute of Environmental Science and Technology (ICTA), Universitat Autònoma de Barcelona, 08193, Barcelona, Spain; ^3^ Archaeology of Social Dynamics (ASD), Institució Milà i Fontanals de Recerca en Humanitats, Consejo Superior de Investigaciones Científicas (IMF-CSIC), C/ Egipcíaques 15, 08001 Barcelona, Spain; ^4^ Grup d'Investigació Prehistòrica (GIP), Departament d'Història, Universidad de Lleida, 25005 Lleida, Spain; ^5^ Central Natural Science Collections, Martin Luther University Halle-Wittenberg, Domplatz 4, 06108 Halle (Saale), Germany; ^6^ Institució Catalana d'Història Natural, Barcelona, Catalonia, Spain; ^7^ Laboratoire d'Anatomie comparée, Ecole Nationale Vétérinaire, de l'Agroalimentaire et de l'Alimentation, Nantes Atlantique – ONIRIS, Nantes Cedex 03, France; ^8^ GEROM, UPRES EA 4658, LABCOM ANR NEXTBONE, Faculté de santé de l'Université d'Angers, Angers, France; ^9^ Institut des Sciences Vétérinaires, Laboratoire « Gestion de la santé et productions animales », Université des frères Mentouri, El Khroub, Algérie

**Keywords:** caprines identifications, GMM, *Ovis aries*, *Capra hircus*, paleoproteins, third lower molar

## Abstract

Geometric morphometrics can effectively distinguish isolated third lower molars of present-day sheep and goat, but its applicability to archaeological specimens has yet to be established. Using a modern reference collection of 743 sheep and goats and a two-dimensional landmark-based geometric morphometric (GMM) protocol, this study aimed to morphometrically identify 109 archaeological specimens, used as case studies, dating from the Late Neolithic to the modern period/era. These morphometric identifications were then compared to molecular identifications via collagen peptide mass fingerprinting, known as Zooarcheology by Mass Spectrometry (ZooMS). ZooMS confirmed the morphometric identifications for 104 specimens, with the five misidentified specimens all morphometrically identified as goat. Modern sheep and goats have larger teeth and distinct shapes compared to their archaeological counterparts, suggesting strong differences between archaeological and modern specimens potentially linked with recent breed improvement or geographical origin of the specimens. In addition, for both species, some of the archaeological dental morphologies do not match with any of our modern references. This study validates the applicability of geometric morphometrics for identifying isolated archaeological sheep and goat teeth. It represents a stepping stone for future, non-destructive, bioarchaeological studies of the two species.

## 1. Introduction

Zooarchaeology by mass spectrometry (ZooMS) is increasingly used in archaeology for reliable taxonomic identification of organic materials [1] such as bones [2], leather [3] or parchment [4]. ZooMS, by using peptide mass fingerprinting of collagen, provides an inexpensive tool for archaeological studies and has been used to distinguish domestic caprine species and to detect their arrival in Africa [5–8], Asia [9,10] and Europe [11,12], and to study distinct farming practices [13–20].

Sheep (*Ovis aries*) and goat (*Capra hircus*) are two important species of agro-pastoral systems in the Mediterranean basin since the Neolithic and were often herded together [21–29]. However, they possess distinct ecological [30,31] and economical properties [32–36], making it of prime interest to establish secure identifications of ancient specimens before studying the two species separately. Criteria for distinguishing sheep and goat among archaeological assemblages exist, notably from post-cranial bones [37–39] and have greatly improved the ability to differentiate the two species. On teeth, discrete criteria have been proposed [31,40–45], but none has been universally used for identifying isolated lower third molars that are often found among archaeological assemblages, since, unlike bones, they are better preserved [46], but also often left unidentified because of the lack of secure identification criteria.

Geometric morphometrics (GMM) is more and more used in bioarchaeology [47]. This series of analytical tools is based on the multivariate statistical analysis of set of Cartesian coordinate data allowing us to explore in-depth morphometric variation [48]. Recently, such approaches have been used to quantify sheep and goat morphometric variation based on the size and shape of their lower third molar [49]. This study demonstrated the ability to identify adult modern specimens of the two species with a probability of 93.3%. In addition, the identification of Middle Ages specimens from the site of Missignac (Aimargues, southern France), used as a first case study, appeared congruent with the initial zooarchaeological faunal analyses and interpretations [50,51]. However, in order to assess whether the same geometric morphometrics (GMM) protocol can be used to identify ancient sheep and goat teeth originating from a broader spatio-temporal scale, it is necessary to validate the approach using securely identified archaeological specimens. Archaeological teeth can be found either enclosed in mandibular bone that possess distinct features between the two species [40,43], or isolated (i.e. without the bone), which make their identification particularly challenging even if some discrete criteria have been proposed for both mandible and teeth [31,40–45]. Being able to provide reliable identification is therefore of prime interest to bioarchaeological studies.

Here we aim to (1) identify archaeological specimens based on their third molar shape using geometric morphometrics (GMM), (2) compare those morphometric identifications with newly generated molecular Mass Spectrometry (ZooMS) identifications, and (3) compare the size and shape morphometric variation of modern sheep and goat with their securely identified archaeological counterparts.

## 2. Material and methods

Third lower molars of 743 modern specimens, corresponding to 521 sheep and 222 goats of various breeds and geographical origins from Western Eurasia, including western Europe and north Africa, were used as a reference for the geometric morphometrics analysis (electronic supplementary material, table S1). Part of these specimens were already included in a previous study [49]. Only one tooth per specimen was considered. All studied specimens were adults, with fully erupted third lower molars. Tooth wear stages were used to estimate the age at death of the specimens following the classification established by Payne (1973, 1985) [42,52] and they were ranging from stages 0 to 17 G (i.e. from 1–2 years to more than 8 years). Sheep and goat display distinct sexual dimorphism and changes through age based on their third molar morphometry, but both factors were found to have little impact on the species differentiation [49].

**Figure 1.**
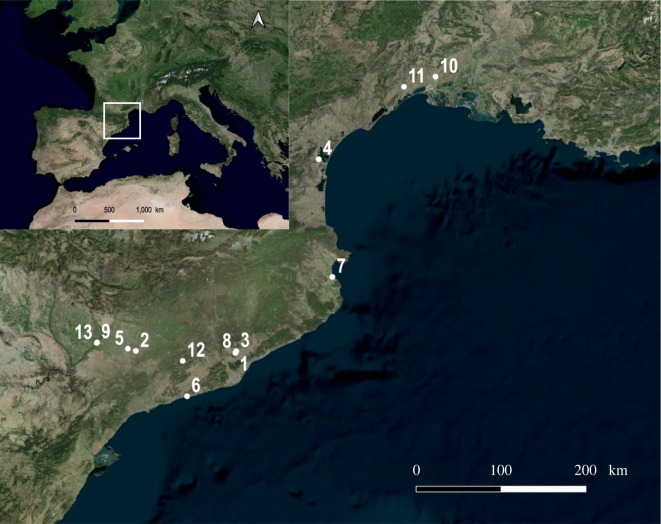
Location of the 13 archaeological sites of the sampled specimens. The numbers refer to the column ID of table 1.

In addition, 109 individuals (electronic supplementary material, table S2) from 13 archaeological sites, selected for their chronological and spatial diversity, dated from the Neolithic to Modern period and located in the north-western part of the Mediterranean basin (present-day Catalonia region in Spain and Occitanie region in Southern France), were used to compare GMM and ZooMS identifications (figure 1, table 1). The selected archaeological specimens consisted of third lower molars with at least a small portion of adherent mandibular bone. For each specimen, the size and shape of the third lower molar were quantified through geometric morphometrics and used for morphometric identification, and a fragment of mandibular bone (normally between 10 to 50 mg) was sampled for molecular ZooMS identification.

**Table 1.** Description of the 13 archaeological sites sampled, indicating geographical origin, chrono-cultural period of occupation, and number of specimens studied by geometric morphometrics and ZooMS.

ID	Country	city	name of the site	chrono-cultural occupation	number of specimens	references
1	Spain	Sant Quirze del Vallès	Bòbila Madurell-Mas Duran	Neolithic	1	[53]
2	Spain	Maldà	Cantorella	Neolithic	1	[54]
2	Spain	Maldà	Cantorella	Bronze Age	1	[54]
3	Spain	Sabadell	Bòbila Madurell - Can Gambús 1	Bronze Age	2	[55]
1	Spain	Sant Quirze del Vallès	Bòbila Madurell-Mas Duran	Iron Age	2	[53]
4	France	Sigean	Pech Maho	Iron Age	15	[56]
5	Spain	Arbeca	Vilars	Iron Age	12	[57]
6	Spain	Calafell	Alorda Park	Iron Age	1	[58]
7	Spain	L'Escala	Empúries	Iron Age	1	[59]
8	Spain	Sabadell	Can Feu	Iron Age	1	[60]
4	France	Sigean	Pech Maho	Iron Age/Antiquity transition	1	[56]
3	Spain	Sabadell	Bòbila Madurell - Can Gambús 1	Antiquity	21	[55]
9	Spain	Lleida	L'Antic Portal de Magdalena	Antiquity	1	[61]
10	France	Aimargues	Missignac	Middle Age	18	[62,63]
11	France	Montpellier	CNR	Middle Age	20	[64]
12	Spain	La Llacuna	Vilademàger	Middle Age	1	[65]
13	Spain	Lleida	La Cuirassa	Middle Age	4	[66,67]
13	Spain	Lleida	La Cuirassa	Modern period	1	[66,67]
5	Spain	Arbeca	Vilars	Out of stratigraphy	4	[57]

### 2.1. Geometric morphometrics

**Figure 2.**
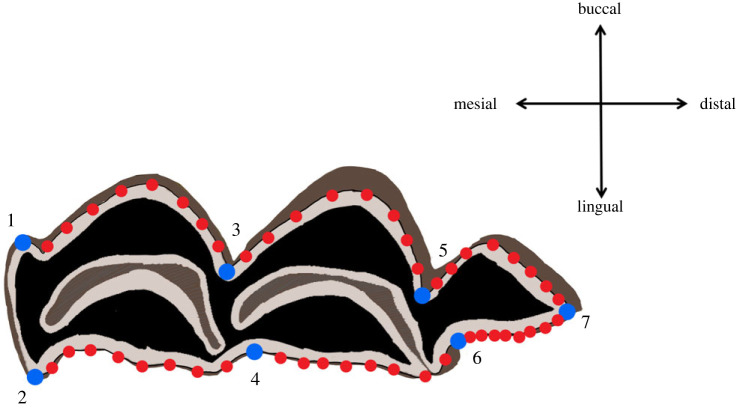
Geometric morphometrics protocol: position of the seven landmarks (in blue) and 48 sliding semi-landmarks (in red) whose coordinates were acquired on a right third lower molar of a sheep. Landmark positions are described in electronic supplementary material, table S2 of [49].

The occlusal view of the third lower molars was photographed using a Nikon d90 LSR camera paired with a 60 mm macro lens (AF-S Micro NIKKOR) attached to a photographic arm (manfrotto 244RC) following a previously published protocol [49]. Right molars were preferentially photographed, and when necessary, photographs of left molars were mirrored prior to GMM data acquisition. Third molar size and shape were quantified using two-dimensional landmarks and sliding landmarks-based geometric morphometrics approaches following the protocol developed in our previous study [49] (figure 2). The coordinates of seven landmarks and of 48 sliding semi-landmarks distributed along six curves (eight equidistant points in each) were acquired using TPSdig2 [68] (for a formal description of the landmark positions see electronic supplementary material, table S2 of [49]) (figure 2). This protocol does not include points along the mesial part of the teeth that is often affected by lateral wear (Jeanjean *et al*., [49]). All data are available in electronic supplementary material, table S3.

### 2.2. Morphometrics identification

All modern and archaeological specimens were superimposed together using a Generalized Procrustes Analysis (GPA) during which the sliding semi-landmarks were allowed to slide by minimizing the sum of the Procrustes distances between each individual and the mean conformation [69–71]. The Procrustes residuals were first analysed using a Principal Component Analysis (PCA) [72–74] (electronic supplementary material, figure S1). A predictive linear discriminant analysis (pLDA), computed on the 20 first PCA scores that maximize the differences between groups [75], was then used to predict to which species the archaeological specimens belonged. Because unbalanced sample size can have a profound effect on discriminant analyses [76], and the fact that sheep greatly outnumber goat in the reference dataset, we used a resampling procedure to down sample the number of sheep to the smaller number of goats, as recommended by previous analysis [77]. The morphometrics identification was therefore based on 100 predictive discriminant analyses (pLDA) performed on two equal-size samples (here 222 in each group) whose results were summarized by the percentage of times a specimen was identified to its correct group [78]. All discriminant analyses were retained and no threshold was fixed for posterior probabilities (i.e. all identifications were based on the 100 pLDA). Only LDA with a cross validation percentage (CVP) above the 3rd quartile of all CVP were retained in order to select the most discriminant analyses. Morphometric identifications were only based on molar shape data, and not size, since it has been previously identified as the most discriminative criteria between sheep and goat [49].

### 2.3. ZooMS

Between 10 and 50 mg of bone was weighed out for each specimen. 250 µl of 0.6 M hydrochloric acid (HCl) was added to each sample then stored at 4°C until being demineralized. Once demineralized, the acid was discarded and three washes of 200 µl each of ammonium bicarbonate (AmBic, NH_2_HCO_2_, pH8) were performed to remove any remaining acid. 100 µl of AmBic was added and the samples were gelatinized at 65°C for 1 h. Samples were centrifuged for 1 minute and 50 µl of the gelatinized supernatant was transferred to a new tube and 0.4 µg µl^−1^ of trypsin was added. The trypsinated samples were digested at 37°C overnight, after which the samples were acidified with 1 µl of 5% trifluoroacetic acid to stop the trypsin. C18 zip tips (Pierce, Thermo Scientific) were used to purify the samples which were eluted in a 50 µl volume. 1 µl of sample topped with 1 µl of matrix (α-cyano-4-hydroxycinnamic acid) was spotted in triplicate along with calibration standards on either a Bruker MTP 384 ground steel MADLI plate and analysed on a Bruker Ultraflex III MALDI-ToF-MS, or onto a SCIEX Opti ToF stainless steel MALDI plate and analysed with an AB SCIEX 4700 MALDI-ToF- MS in the Department of Prehistory & Institute of Environmental Science and Technology (ICTA) at Universitat Autònoma de Barcelona (Barcelona, Spain). Triplicate spectra for each sample were averaged and analysed using mMass [79] and compared to a database of known reference species [2,80–82].

### 2.4. Comparison of ancient and modern sheep and goat

The overall percentage of correct identification was computed comparing the geometric morphometrics and ZooMS identifications. Securely ZooMS-identified archaeological sheep and goat were then compared with their modern counterparts using Wilcox-tests for size and Procrustes Anova (procD.lm function from geomorph package [83]) for shape. Size and shape variation were visualized using boxplot and PCA, respectively. Homogeneity of size and shape difference between species and between archaeological versus modern specimens were tested using 2-way Anova and 2-way Procrustes Anova, respectively.

Morphometrics proximities between modern sheep and goat and the archaeological specimens identified via ZooMS were visualized by a neighbour-joining network computed on Mahalanobis distances. Graphics were created in R using the ‘ggplot2’ package [84]

Change in molar shape and size variance between archaeological and modern specimens of sheep and goat was assessed, respectively, using the disparity test of the geomorph R package [85,86] and Fligner test (stats package [87]).

All the analysis were performed in R v. 6.1.524 [87] through Rstudio v. 4.2.2 [88].

## 3. Results

### 3.1. Identification for archaeological specimens

**Figure 3.**
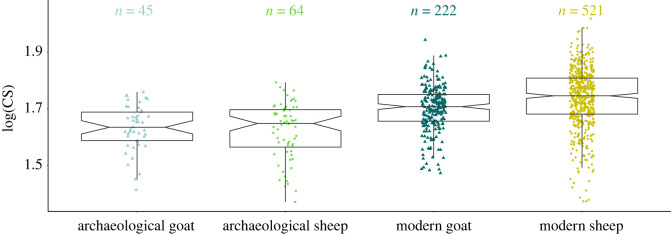
Third lower molar size variation: boxplot showing variation in log centroid size between modern (dark) and archaeological (light) sheep (green) and goat (blue) identified by ZooMS. For differences between sites and chronologies, see electronic supplementary material, figure S2.

Based on their third molar shape, 59 archaeological specimens were identified as sheep (54.1%) and 50 as goat (45.9%) (figure 3). The 109 identified were based on 31 LDA with cross-validation percentages above 94.22% [90% Confidence Interval: 93.01–95.50].

For the same 109 specimens, ZooMS identified 64 (58.7%) specimens as sheep and 45 (41.3%) as goat (all spectra supporting the results are available in the Dryad Digital Repository https://doi.org/10.5061/dryad.fj6q5741m [89]).

The two approaches provided the same species identification for 104 (95.4%) of the archaeological specimens corresponding to 59 sheep and 45 goats. The five (4.6%) remaining specimens are from the sites of Can Gambús (Catalonia, Late Antiquity) (*n* = 3) and Missignac (France, Late Antiquity and Middle Age) (*n* = 2). These misidentified specimens are all morphometrically identified as goat but molecularly identified as sheep. All of these misidentified specimens fell into the most common age category of 4–8 years old (i.e. 11 G) (electronic supplementary material, table S2). Looking more closely at these misidentifications, it appears that three out of the five specimens were identified 100% of the time to goat via GMM (i.e. all LDA give ‘goat’ as identification, no matter the probability of each of the 100 LDAs), while the two last ones were attributed respectively to goat only in 74.2% and 81.8% of the cases (see electronic supplementary material, figure S1 for probability of each LDA).

#### 3.2. Morphometrics comparison with modern specimens

The ancient samples studied were selected to represent a diversity of geographical origins (Catalonia and the South of France) and chrono-cultural periods (Late Neolithic to modern period) but certainly do not cover the entire past sheep and goat diversity in the North Western Mediterranean basin. Sample sizes per region and chrono-cultural period were too small to envision detailed morphometrics comparison and all archaeological specimens were analysed jointly (see electronic supplementary material, figure S2 for a visualization of size differences between sites and chrono-cultural periods). Overall, the archaeological sheep and goat possessed smaller third lower molars than their modern relatives (figure 3, table 2). Modern sheep also appear larger than goat, contrary to the pattern observed for archaeological specimens (figure 3, table 2).

**Table 2.** Differences in size between modern sheep and goat, and the archaeological specimens identified by ZooMS. Pairwise Wilcoxon test were done between each group. The *p*-values in bold are significant (*p*-value < 0.05) after Bonferroni correction for multiple comparisons.

	archaeological goat	archaeological sheep	modern goat
archaeological sheep	1		
modern goat	3.9 × 10^−6^	1.4 × 10^−7^	
modern sheep	9.6 × 10^−11^	2.9 × 10^−14^	9.1 × 10^−8^

**Figure 4.**
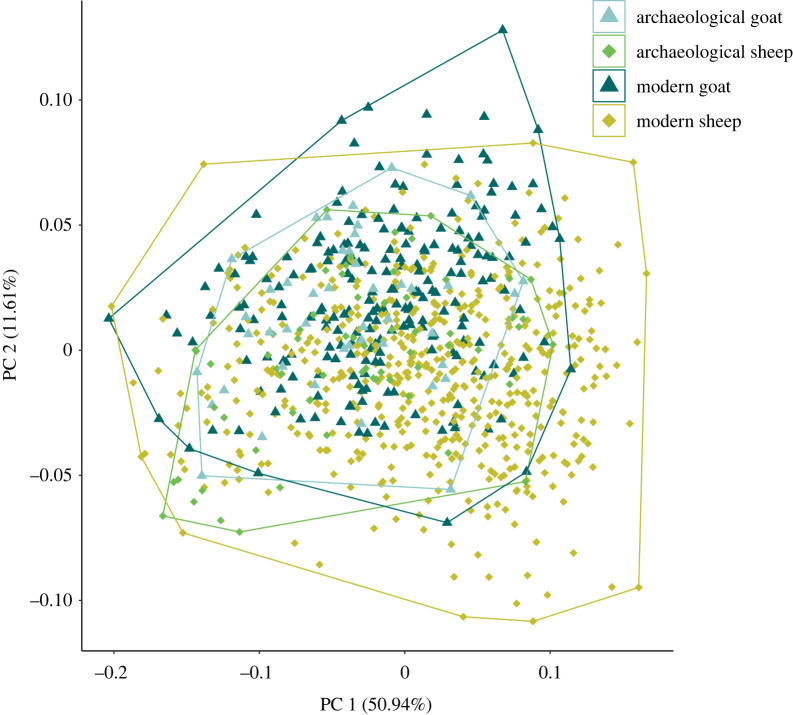
Two first axes of the PCA showing variation between modern (dark) and archaeological (light) sheep (green) and goat (blue) identified by ZooMS. PCA for each species are available in electronic supplementary material, figure S3.

A PCA of all archaeological and modern sheep and goat shows that the modern specimens studied show new morphologies that didn't exist in the past (figure 4).

**Figure 5.**
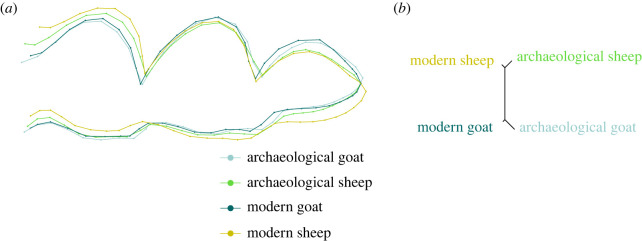
Visualization of the mean shape (*a*) and dissimilarity network (*b*) between archaeological (light) and modern (dark) sheep (green) and goat (blue). Archaeological specimens are identified by ZooMS.

Shape differences between sheep and goat are present for both modern and archaeological specimens (respectively *p*-value = 0.013 and *p*-value = 0.001). Mean shape visualization and dissimilarity network on shape confirm that there is a higher proximity within species than within periods, that is to say the archaeological goat (identified through ZooMS) reassemble more to modern goat than to archaeological sheep (and conversely) (figure 5), even if there are shape differences between archaeological and modern sheep (*p*-value = 0.001) and goat (*p*-value = 0.004).

In addition, differences between sheep and goat are homogeneous between modern and archaeological specimens for both shape (interaction term of a 2-way Procrustes ANOVA, *p*-value = 0.463) and size (*p*-value = 0.138).

The only difference in terms of size or shape variance is seen between modern sheep and goat, with modern sheep showing more variation than modern goat (table 3).

**Table 3.** Comparison of size and shape variance between modern and archaeological sheep and goat. Size variance was tested with the Fligner test, with a Khi^2^ test statistic, and shape variance with a disparity approach (pairwise test on Procrustes variance, distance between each Procrustes variance).

	size		shape	
	Khi^2^	*p*-value	distance	*p*-value
archaeological sheep/archaeological goat	2.34	0.12	1,71 × 10^−3^	0.26
modern sheep/modern goat	15.41	8.63 × 10^−5^	2,07 × 10^−3^	0.002
archaeological sheep/modern sheep	3.64 × 10^−4^	0.98	1,69 × 10^−3^	0.11
archaeological goat/modern goat	0.11	0.73	1,34 × 10^−3^	0.31

## 4. Discussion

We demonstrate, for the first time, that geometric morphometrics can be an efficient alternative to molecular identification with 95.4% of the morphometric identifications being congruent with those provided by the molecular ZooMS identification.

Identification of paleontological or archaeological remains is often done by actualism, defined as ‘the methodology of inferring the nature of past events by analogy with processes observable and in action in the present’ [90]. In order to fully explain the morphological diversity in the past, a better understanding of current diversity and its incorporation in the analyses is needed [91,92]**.** The hypothesis based on these identifications assumed that modern specimens are relevant for studying remains from the past, with negligible influence of extrinsic (e.g. temporal or geographical) variation. Modern specimens are often the only available reference of securely identified species, however, it has been demonstrated that modern references can lead to misidentifications. For example, modern dog breeds are not a good proxy to identify archaeological specimens [93]. This is also the case for other domestic species for which it is well known that direct selection over the last two centuries was intensified, followed by standardization of morphology and performance with the emergence of the breed concept [94–97]. This also applies to sheep and goat, whose breeds became genetically more and more uniformized [95], even if our morphometric results do not detect increase in diversity between our ancient and modern datasets. However, we detected morphologies that exist today that were not present in the studied archaeological dataset. This result will have to be explored further using more archaeological specimens.

We found that a large majority (95.4%) of the specimens were correctly identified based on their third molar shape, suggesting that the morphometric integrity, and thus the distinction of the species, are maintained through time, at least for the third lower molar. This means that, based on their third molar shape, the differences between sheep and goat remain the same through time. On the other hand, modern specimens of both species possess larger teeth than their archaeological counterparts with a strong overlap between sheep and goat, confirming the impossibility of using size alone for species identification. This size difference between archaeological and modern specimens, although little studied, has also been suggested for cattle [98] and pigs [99].

The five misaligned specimens, those identified as sheep by ZooMS and goat by geometric morphometrics, suggest that some ancient sheep dental morphologies were more similar to the here-captured present-day goat dental morphologies. It seems important to note that the percentage of correct identification of 95.4% observed here is similar to the 93.3% percentage of correct cross-validation between modern sheep and goat obtained in a previous study [49]. At least four hypotheses could be envisaged to explain the misidentified specimens.

First, even if our modern reference collection includes 521 sheep and 222 goats belonging to a range of breeds originating from Europe and North Africa, the entire existing modern diversity is certainly not represented. Furthermore, this reference collection was purposely built to study the north western Mediterranean region, and so may not be directly suitable for other geographical areas. Similarly, two waves of exotic foods are known during Iron Age [100], the Roman and medieval periods [101,102] that may have influenced the diversity we observe in our archaeological dataset. Consequently, we cannot exclude that these five individuals may reflect animal translocation across regions, from areas where sheep dental morphology was similar to that of present-day goats.

Second, these animals could correspond to taxa not included in our analyses—e.g. the mouflon (*Ovis orientalis musimon,* a feral form of sheep), the alpine (*Capra ibex*) or Iberian ibex (*Capra pyrenaica*) or the Barbary sheep (*Ammotragus lervia*)—whose dental morphology and geographical distributions in the past are largely unknown, and whose distinction based on collagen peptide sequences is limited. Due to the highly conserved nature of the amino acid sequence of collagen, ZooMS cannot be used to distinguish between domestic and wild specimens of the same species, and sometimes cannot distinguish between closely related taxa, as their collagen sequences are identical or near identical [80]. For example, domestic sheep, mouflon and Barbary sheep cannot be distinguished from another using ZooMS as their collagen peptide markers are the same. It is possible that these five samples belong to taxa that are closely related to domestic sheep however have dentition more similar to that of domesticated goats. This hypothesis could be tested in the future through e.g. palaeogenomics.

Third, age and sex are known to have a significant impact on sheep and goat third molar morphology, but that both factors have limited impact on the ability to discriminate the two species [49]. Indeed, even if age (estimated through wear stages) affects the third lower molar shape of both species, a closer proximity between species than between age categories has been found [49]. In addition, it was demonstrated that the impact of age on the between species differentiation was mostly due to the oldest specimens (more than 8 years) [49]. Because the mis-indentitied specimens had an estimated age of 4–8 years, it seems unlikely that age caused the mismatch between the morphometric and molecular identifications. Sexual dimorphism in teeth size and shape has been identified in both species; but as for age, a greater proximity has been found between species than sex, and the sexual dimorphism has no impact on modern caprine identification [49].

Finally, even if very rare, sheep-goat hybrids exist (e.g. [103,104]). Such hybrids would likely be difficult, if not impossible, to identify using either ZooMS or GMM, as presumably the results for both analyses would simply indicate either sheep or goat, rather than a mix.

Nevertheless, with a approximately 95% success rate, this newly generated data allows us to build a secure reference collection of ancient morphologies for the two species from the Neolithic to Modern periods in the North Western Mediterranean basin. This will enable non-molecular, and thus non-destructive, methods of analysis of dentition for larger zooarchaeological assemblages to improve our understanding of the husbandry history of these two species in space and time. Because this study aims only at validating the methodological approach, the archaeological interpretations require further consideration. While the number of specimens studied here did not allow detailed analyses, adding more specimens will allow further exploration of the environmental and socio-cultural factors that shaped the past agrobiodiversity and its evolution. While it is known that modern [31] and ancient goat and sheep have different diets [105], the impact of diet in tooth morphology in those species remains to be explored, but in any case did not have a major impact on the taxonomic identification.

The observed size and shape differences between archaeological and modern specimens of both sheep and goats are in agreement with previous zooarchaeological results. Here we observed that archaeological specimens have on average smaller molars than their modern counterparts. Sheep and goat are known to have increased in size during Modern period [106–108]. Caprines were particularly improved upon over the last centuries [108–110], which could explain the differences noticed between archaeological and modern groups. However, despite the presence of both primitive and standardized breeds in our referential, we did not find that modern specimens were more or less diverse than the archaeological groups. The only observed variance difference is between modern sheep and goat, where modern sheep are, for both size and shape, more variable than modern goat. This result could be due to the greater number of sheep compared to goat in the modern referential. Moreover, today officially recognized sheep breeds are also more numerous than goat, with for example around 50 different French sheep breeds compared to only 15 breeds of goat [97,111]. Moreover, goats were mainly selected for the purpose of milk, while sheep have been selected for meat, milk and wool [97,111], which could explain this greater morphological diversity, and the greater number of sheep breeds compared to goat.

Archaeological sheep and goat dental remains can prove difficult to identify to the species level [38,40,42,43,112,113]. This study is a new contribution toward analyses dedicated to understanding the evolution of both sheep and goat in archaeological records using non-destructive morphometric identification of dental specimens. Being able to identify isolated third molars, that often remain unidentified in archaeozoology, allows new perspectives for further research. It is now possible to use the powerful and non-invasive quantitative tools of geometric morphometrics, via archaeophenomics [47], to explore in more depth the separate evolution of sheep and goat through time, and follow their socio-economic and cultural roles in past animal husbandry. Such studies require secure identification and quantification which, particularly for large scale assemblages, is best accomplished through integrating molecular and non-molecular methodologies. Integrative approaches that combine GMM and biomolecular methods, such as ZooMS, are in high demand, with such integration leading to clearer perceptions of domestic species evolution through time and space.

## Data Availability

The data are provided in electronic supplementary material [114].
